# Quantification of intramuscular fat in patients with late-onset Pompe disease by conventional magnetic resonance imaging for the long-term follow-up of enzyme replacement therapy

**DOI:** 10.1371/journal.pone.0190784

**Published:** 2018-01-09

**Authors:** André Lollert, Clemens Stihl, Andreas M. Hötker, Eugen Mengel, Jochem König, Katharina Laudemann, Seyfullah Gökce, Christoph Düber, Gundula Staatz

**Affiliations:** 1 Department of Diagnostic and Interventional Radiology, Section of Pediatric Radiology, Medical Center of the Johannes Gutenberg University, Mainz, Germany; 2 Department of Diagnostic and Interventional Radiology, Medical Center of the Johannes Gutenberg University, Mainz, Germany; 3 Villa Metabolica, Center of Pediatric and Adolescent Medicine, Medical Center of the Johannes Gutenberg University, Mainz, Germany; 4 Institute for Medical Biostatistics, Epidemiology and Informatics, Medical Center of the Johannes Gutenberg University, Mainz, Germany; Azienda Ospedaliero-Universitaria Santa Maria della Misericordia, ITALY

## Abstract

**Objective:**

The objective of this study was to evaluate a quantitative method based on conventional T1-weighted magnetic resonance (MR) imaging to assess fatty muscular degeneration in patients with late-onset Pompe disease and to compare it with semi-quantitative visual evaluation (the Mercuri score). In addition, a long-term retrospective data analysis was performed to evaluate treatment response to enzyme replacement therapy with alglucosidase alfa.

**Methods:**

MR images of the lumbar spine were acquired in 41 patients diagnosed with late-onset Pompe disease from 2006 through 2015. Two independent readers retrospectively evaluated fatty degeneration of the psoas and paraspinal muscles by applying the Mercuri score. Quantitative semi-automated muscle and fat tissue separation was performed, and inter-observer agreement and correlations with clinical parameters were assessed. Follow-up examinations were performed in 13 patients treated with alglucosidase alfa after a median of 39 months; in 7/13 patients, an additional follow-up examination was completed after a median of 63 months.

**Results:**

Inter-observer agreement was high. Measurements derived from the quantitative method correlated well with Medical Research Council scores of muscle strength, with moderate correlations found for the 6-minute walk test, the 4-step stair climb test, and spirometry in the supine position. A significant increase in the MR-derived fat fraction of the psoas muscle was found between baseline and follow-up 1 (*P* = 0.016), as was a significant decrease in the performance on the 6-minute walk test (*P* = 0.006) and 4-step stair climb test (*P* = 0.034), as well as plasma creatine kinase (*P* = 0.016). No statistically significant difference in clinical or MR-derived parameters was found between follow-up 1 and follow-up 2.

**Conclusions:**

Quantification of fatty muscle degeneration using the semi-automated method can provide a more detailed overview of disease progression than semi-quantitative Mercuri scoring. MR-derived data correlated with clinical symptoms and patient exercise capacity. After an initial worsening, the fat fraction of the psoas muscle and performance on the 6-minute walk test stayed constant during long-term follow-up under enzyme replacement therapy.

## Introduction

Late-onset Pompe disease (LOPD) is a rare, inherited, autosomal-recessive condition, characterized by a lack of lysosomal acid alpha glycosidase. This enzyme deficiency results in the intralysosomal accumulation of glycogen in multiple body tissues, including skeletal muscle [1]. Although the pathophysiology of muscle damage is not yet completely understood, lysosomal enlargement and rupture, as well as dysfunctional autophagy, lead to replacement of muscle tissue by fat [2]. Patients clinically present with progressive proximal weakness, particularly of their trunk and limb muscles, and respiratory insufficiency or failure [3]. Though less frequently assessed in the literature, the trunk muscles are highly relevant to characterizing the disease. In fact, these muscles were identified in a recent electromyogram-based study having the most frequent pathologies [4]. In contrast with patients who have infantile-onset Pompe disease with mortality during the first year of life in most cases, patients with LOPD are usually diagnosed during adolescence or adulthood, and present with a much slower disease progression [1]. Nevertheless, both populations can benefit from treatment.

Modern treatments for LOPD include enzyme replacement therapy (ERT) using recombinant alglucosidase alfa, which can stabilize or delay muscular degeneration [5,6]. Treatment efficacy is primarily monitored using clinical strength tests and pulmonary function tests [5,7]. Although morphologic changes in the musculature can be evaluated invasively by biopsy, non-invasive imaging also has the potential, particularly during follow-up, to provide a more accurate and objective clinical evaluation, and to identify clinically silent changes in the musculature [8].

These changes can be assessed by computed tomography [9] but the downside to this approach is the need for ionizing radiation. The current clinical standard for evaluating the extent of fatty muscle degeneration is to use magnetic resonance imaging (MRI) [10,11]. In the past, a visual, semi-quantitative scoring system [12] (the Mercuri score) was applied to standard T1-weighted spin echo sequences [13] to assess fatty muscle degeneration. This score is visually applied by the radiologist and ranges from 1 (normal muscle appearance) to 4 (severe involvement, more than 60% fatty degeneration). More recently, new MRI techniques have been developed to separate water and fat signals [14]. Dixon (water-/fat-) sequences have been used to quantify fatty muscle infiltration in LOPD patients [15], with a significant advantage being their robustness against B0-/B1-inhomogeneities of the magnetic field. Another promising technique is proton-density measurement of the fat fraction (FF) [16], but this technique has not yet been used for the assessment of LOPD patients. These techniques are appropriate for the monitoring of contemporary and future cases, but older MRI data may comprise only conventional spin echo (SE) sequences, complicating their comparison with recent data.

The assessment of fatty muscle degeneration by MRI using grayscale values based on signal intensity has been reported using T1-weighted images [17]. Another group recently introduced a quantification method based on signal intensity measurements of the paraspinal muscles in relation to a small encircled area of subcutaneous fat from the same level [18]. A semi-automated quantitative approach based on T2-weighted SE sequences has also been proposed for the evaluation of fatty degeneration of paraspinal muscles [19], but this method has not yet been applied on muscle degeneration arising from metabolic disorders.

Tthe purpose of this study therefore was to evaluate inter-observer agreement of this latter quantitative method based on conventional T1-weighted MRI for the assessment of fatty muscular degeneration in patients with LOPD compared with Mercuri scores. We also assessed correlations with clinical parameters, and used the proposed quantitative method to evaluate long-term follow-up under ERT.

## Materials and methods

### Patients

#### Initial MR evaluation

The initial MRI examinations of 41 patients (22 men, 19 women, median age: 28 years, range: 6–74 years) with confirmed LOPD presenting at the metabolic diseases clinic (“Villa Metabolica”) of the Medical Center of the Johannes Gutenberg University, Mainz, Germany, between 2006 and 2015 were analyzed in this retrospective single-center study. All patients provided written informed consent to the MRI examination and data evaluation. Approval by the local independent ethics committee was waived because of the retrospective study design.

#### Follow-up under ERT

Follow-up MRI examinations were available in 21 patients. Of these, one patient was excluded because the follow-up examination was less than 24 months after the initial MRI; five more patients were excluded because ERT was not initiated at all (n = 1; this patient was evaluated separately to demonstrate natural disease progression), no information concerning ERT could be retrieved from the medical record (n = 3), or ERT was initiated more than 24 months after the baseline MRI (n = 1). Two more patients were excluded, because ERT had been initiated one and two years prior to the first MRI examination. The remaining 13 patients were eligible for the assessment of long-term follow-up under ERT. Detailed characteristics of these patients are shown in S1 Table. All patients were treated according to a standardized scheme with 20 mg alglucosidase alfa (Myozyme®, Sanofi-Genzyme) per kilogram of body weight every 2 weeks. The first follow-up MRI was performed at a median of 39 months (range 24 to 50 months) after the baseline examinations. Seven patients had a second follow-up MRI at a median of 63 months (range 50 to 83 months) after the initial examination.

### Muscle MRI

Examinations were performed using 1.5 T (Magnetom Avanto®, Siemens, Erlangen, Germany; 45 mT/min, slew rate = 200 T/m/ms, n = 51) or 3T (Magnetom Skyra®, Siemens, Erlangen, Germany; 45 mT/min, slew rate = 200 T/m/ms, n = 14) systems. The integrated spine array coil was used for signal detection.

The imaging protocol consisted of T1-weighted turbo spin echo (TSE) sequences of the lumbar spine in the coronal and transverse orientations. All sequences were acquired using a reconstruction matrix of 384x384, a slice thickness of 5 mm, and a flip angle of 150°. Other scan parameters at 1.5 T were as follows: repetition time / echo time (TR/TE) 600/12 ms, echo train length (ETL) 4, and field of view (FoV) 280 mm; at 3T these parameters were as follows: TR/TE 630/11 ms, ETL 2, and FoV 200 mm.

### Semi-quantitative evaluation of imaging data (the Mercuri score)

Two radiologists (respectively with 6 and 8 years of experience in MRI reading) independently evaluated imaging data. Mercuri scores [12] were determined to visually assess the extent of fatty degeneration of the psoas and paraspinal muscles.

### Quantitative semi-automated evaluation

**Fig 1:**
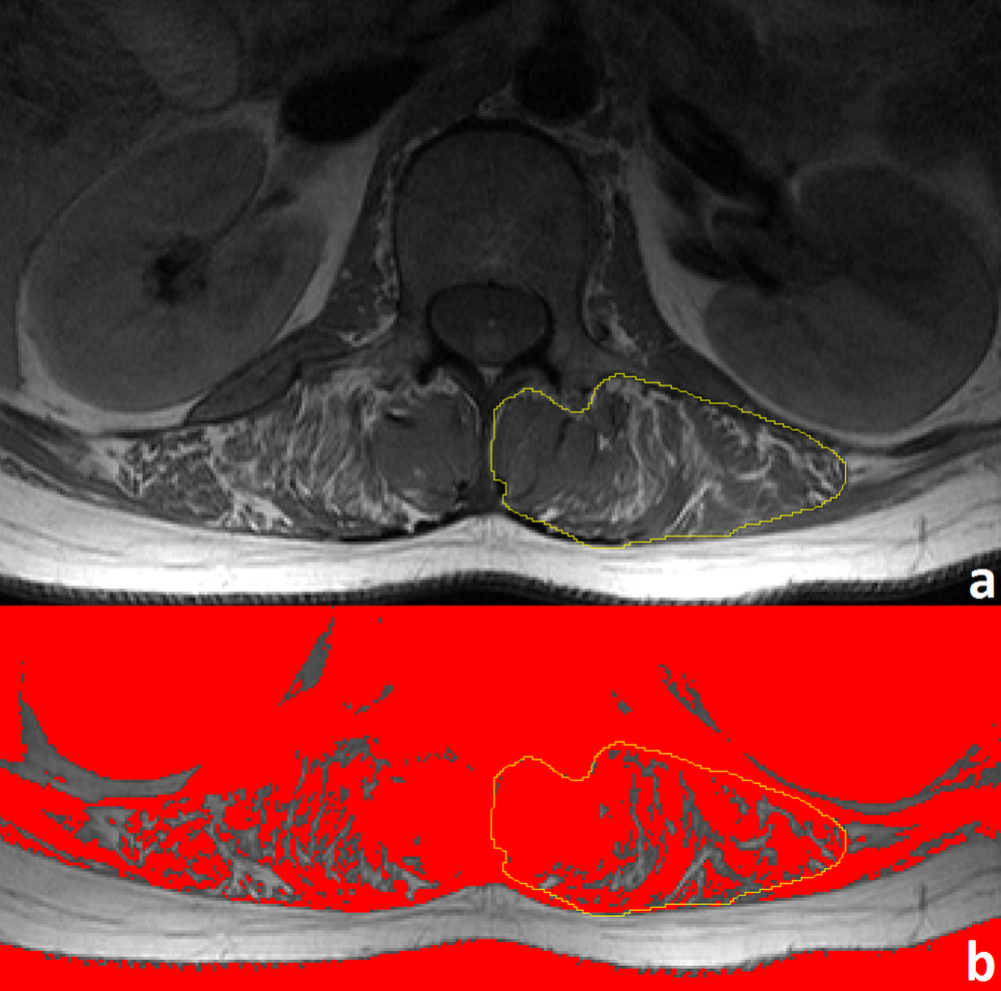
MR-derived measurement of the fat fraction. A region of interest is selected from a T1-weighted TSE sequence in the left paraspinal musculature at the L3-level (panel a). Fat fraction is calculated using the equation FF = 1 –FCSA/CSA; a signal intensity threshold of 772 (derived from normal muscle–the medial multifidus muscle in this case) segregates muscle from fat tissue. Pixels beneath this threshold, colored red (panel b), constitute the FCSA (1096 mm^2^), while the entire area of the ROI constitutes the CSA (1466 mm^2^). These data result in an FF value of 0.25.

All measurements were performed by two independent observers using freely available image analysis software (ImageJ, version 1.49; U.S. National Institutes of Health, Bethesda, Maryland, USA; http://rsb.info.nih.gov/ij). Bilateral regions of interest (ROIs) were defined using transverse slices at the L3- and L5-levels for both the psoas and paraspinal muscles, with the latter including the spinotransverse muscles (predominantly multifidus) and erector spinae group (longissimus and iliocostalis) [20]. For each ROI, several parameters were calculated, as proposed by Fortin and Battié [19]. These parameters included the total cross-sectional area (CSA) and functional cross-sectional area (FCSA), with the latter providing a measure of the remaining functional tissue in the respective muscles. In summary, FCSA measurements are governed by a thresholding method in which the upper limit for mean muscle tissue signal intensity is defined by the reader. Fat fraction was defined by the equation FF = 1 –FCSA/CSA (Fig 1). Resulting values ranged from 0 (no degeneration) to 1 (complete fatty degeneration). The total time required for these evaluations was approximately 10 minutes per case. For the assessment of the follow-up examinations, mean values of FF (including left- and right-sided ROIs at the L3- and L5-levels) were calculated for the psoas and paraspinal muscles.

### Clinical examinations

An assessment of clinical severity was made on the day of the MRI examination. Several tests were performed: a 6-minute walk test [21]; the 4-step stair climb test [22]; spirometry, with assessment of the forced vital capacity (in the sitting and supine positions); and strength tests of the paraspinal and psoas muscles [23]. This latter test used the Medical Research Council (MRC) scale for muscle strength (only full grades were used), with the psoas muscle tested bilaterally, and the lower score taken if discordant. Laboratory tests included levels of plasma creatine kinase (CK).

### Statistics

Inter-observer agreement for Mercuri scores was assessed using linear-weighted kappa (κ). A κ coefficient of >0.6–0.8 indicated good agreement, with an almost perfect agreement indicated by a κ of between 0.81 and 1. Inter-observer agreement for FF was assessed using the two-way mixed single-measure intra-class correlation coefficient (ICC). ICCs of > 0.75 or > 0.9 indicated a good and excellent agreement, respectively [24]. Correlations between Mercuri scores and clinical parameters were tested for significance using the Kruskal-Wallis test. Correlations between Mercuri scores, as well as FF and clinical parameters were quantified by Spearman’s correlation coefficient (ρ). Differences in clinical and MR-parameters during follow-up were tested for significance by applying the Wilcoxon-test. A *P* value < 0.05 was considered significant in all statistical analyses, which were exploratory; no adjustments of *P* values for multiple tests were performed, and the presented *P* values therefore are descriptive. All data analyses were carried out with SPSS Statistics (version 23.0, IBM, Ehningen, Germany).

## Results

### MRI and inter-observer agreement

The initial MRI examinations could be analyzed in all 41 patients. Mercuri scores as assessed by the two observers are shown in Table 1. Inter-observer agreement for Mercuri scoring was good, both for the psoas (κ = 0.89 [95% confidence interval 0.81–0.97]) and paraspinal muscles (κ = 0.85 [95% confidence interval 0.76–0.94]).

**Table 1:** Inter-observer agreement of the Mercuri scores in the psoas and paraspinal muscles.

**psoas muscle**		**observer 2**			
		**M1**	**M2**	**M3**	**M4**
**observer 1**	**M1**	20	0	0	0
	**M2**	1	4	2	0
	**M3**	0	1	2	2
	**M4**	0	0	0	9
**paraspinal muscles**		**observer 2**			
		**M1**	**M2**	**M3**	**M4**
**observer 1**	**M1**	14	0	0	0
	**M2**	5	2	1	0
	**M3**	0	0	2	3
	**M4**	0	0	0	14

Numbers indicate the total number of patients assigned to the respective Mercuri score (M1-M4) by both observers.

Mean FFs of the psoas muscle, as assessed by observer 1, were 0.37 (± 0.35) at the L3-level, and 0.35 (± 0.37) at the L5-level (*P* = 0.515).Means for FFs of the paraspinal muscles were 0.42 (± 0.38) at the L3-level, and 0.43 (± 0.35) at the L5-level (*P* = 0.617).

Mean FFs of the psoas muscle, as assessed by observer 2, were 0.34 (± 0.37) at the L3-level, and 0.37 (± 0.39) at the L5-level (*P* = 0.277). Means for FFs of the paraspinal muscles were 0.47 (± 0.4) at the L3-level, and 0.49 (± 0.36) at the L5-level (*P* = 0.124).

**Fig 2:**
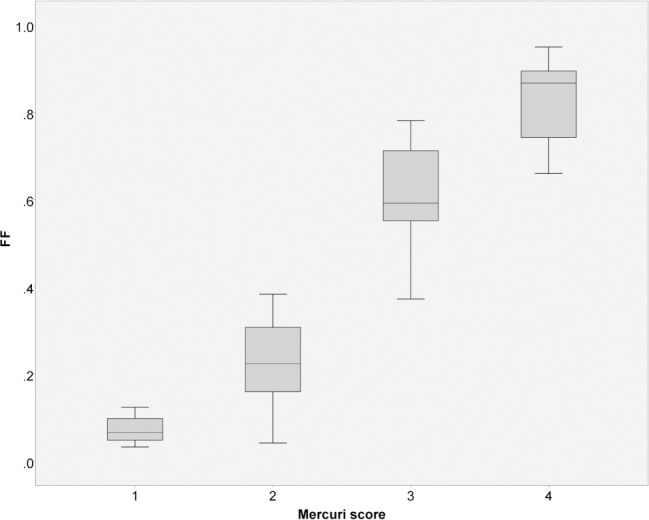
Representative correlates between the Mercuri scores for paraspinal muscle and FF. Data derived from observer 1 at the L5-level, Spearman’s ρ = 0.92, *P* < 0.001.

FF correlated well with Mercuri scores, although some overlap of quantitative data was seen for adjacent Mercuri scores (Fig 2).

Inter-observer agreement was high for the semi-automated method with ICCs indicating an almost perfect agreement. Inter-observer differences mainly occurred with a depiction of the relatively small cross-sectional areas of the psoas muscle at the L3-level (Table 2).

**Table 2:** Inter-observer agreement concerning quantitative MR-derived data.

Localization	CSA	FCSA	FF
psoas L3 right	0.617 [0.388–0.775]	0.742 [0.565–0.853]	0.931 [0.876–0.963]
psoas L3 left	0.761 [0.593–0.865]	0.843 [0.725–0.913]	0.933 [0.878–0.964]
paraspinal L3 right	0.895 [0.813–0.943]	0.959 [0.887–0.982]	0.968 [0.898–0.986]
paraspinal L3 left	0.875 [0.778–0.931]	0.959 [0.892–0.982]	0.971 [0.925–0.987]
psoas L5 right	0.914 [0.846–0.953]	0.964 [0.964–0.98]	0.97 [0.944–0.984]
psoas L5 left	0.832 [0.706–0.906]	0.919 [0.854–0.956]	0.96 [0.927–0.979]
paraspinal L5 right	0.823 [0.428–0.929]	0.923 [0.526–0.975]	0.953 [0.822–0.981]
paraspinal L5 left	0.874 [0.72–0.939]	0.937 [0.694–0.977]	0.954 [0.82–0.981]
**total average**	**0.824 [0.659–0.905]**	**0.906 [0.763–0.952]**	**0.955 [0.886–0.978]**

Two-way mixed single-measure intra-class correlation coefficients (ICC) with 95% confidence intervals for CSA, FCSA, and fat fraction (FF = 1-FCSA/CSA) are given, *P* value is < 0.001 for all coefficients.

CSA = cross-sectional area

FCSA = functional cross-sectional area

### Correlates between MRI and clinical data

Mercuri scores (with categories 2 and 3 combined because of low abundance) for both observers correlated well with clinical parameters, especially for the 6-minute walk test, forced vital capacity in the supine position, and MRC scores (Table 3). Plasma CK levels were significantly lower in patients with a very high degree of fatty muscle degeneration (Mercuri score 4) compared to low to intermediate degeneration (Mercuri scores 1–3, Table 3). Correlations between Mercuri scores, and MR-derived FF and clinical data are also shown in Table 3. For most of the assessed clinical tests, correlation coefficients with the FFs were slightly higher compared to correlation coefficients with the Mercuri scores.

**Table 3:** Correlations between MR-derived data and clinical parameters.

	6-minute walk test(n = 39)	4-step stair climb test(n = 36)	FVC sitting (n = 40)	FVC supine (n = 40)	creatine kinase (n = 41)	MRC score (n = 40)
**psoas muscle**						
Mercuri 1	535 m	2.1 sec	3.2 l	3 l	786 U/l	5
Mercuri 2/3	473 m	3 sec	3.2 l	2.7 l	895 U/l	3
Mercuri 4	332 m	3.7 sec	2.4 l	1.4 l	405 U/l	2
*P* value	**0.049**	0.066	0.172	**0.006**	0.114	**<0.001**
**paraspinal muscles**						
Mercuri 1	583 m	2.1 sec	3.1 l	3 l	993 U/l	5
Mercuri 2/3	411 m	2.5 sec	3.3 l	2.4 l	892 U/l	4
Mercuri 4	371 m	3.3 sec	2.6 l	2.2 l	388 U/l	4
*P* value	**0.025**	0.064	0.387	0.068	**0.007**	**0.004**
**psoas muscle**						
ρ with Mercuri (*P* value)	-0.4 (**0.012**)	0.39 (**0.018**)	-0.3 (0.062)	-0.52 (**0.001**)	-0.28 (0.079)	-0.68 (**<0.001**)
ρ with FF L3 (*P* value)	-0.42 (**0.008**)	0.43 (**0.009**)	-0.35 (**0.027**)	-0.56 (**<0.001**)	-0.19 (0.226)	-0.59 (**<0.001**)
ρ with FF L5 (*P* value)	-0.48 (**0.002**)	0.51 (**0.002**)	-0.37 (**0.019**)	-0.56 (**<0.001**)	-0.26 (0.103)	-0.63 (**<0.001**)
**paraspinal muscles**						
ρ with Mercuri (*P* value)	-0.4 (**0.012**)	0.37 (**0.025**)	-0.2 (0.22)	-0.37 (**0.019**)	-0.35 (**0.023**)	-0.57 (**<0.001**)
ρ with FF L3 (*P* value)	-0.52 (**0.001**)	0.45 (**0.006**)	-0.26 (**0.1**)	-0.41 (**0.009**)	-0.31 (**0.048**)	-0.6 (**<0.001**)
ρ with FF L5 (*P* value)	-0.52 (**0.001**)	0.5 (**0.002**)	-0.36 (**0.022**)	-0.5 (**0.001**)	-0.28 (**0.032**)	-0.61 (**<0.001**)

Median values of the clinical tests depending on the Mercuri scores of the psoas and paraspinal muscles are presented. *P* values are the result of applying the Kruskal-Wallis test. Additionally, correlations between the Mercuri scores as well as FFs at the L3- and L5-levels with clinical data are described by Spearman correlation coefficients (ρ). Data result from observer 1 (corresponding data as assessed by observer 2 can be derived from S1 Dataset).

FVC = forced vital capacity

MRC = Medical Research Council

### Follow-up under ERT

Clinical data and MR-derived FF values obtained during follow-up are presented in Table 4. A significant decrease in the performance on the 6-minute walk test and 4-step stair climb test was found between baseline and follow-up 1, with no further decrease between follow-up 1 and follow-up 2. A significant decrease in plasma creatine kinase was also shown during follow-up. Corresponding to these clinical findings, the MR-derived FF of the psoas muscle increased significantly between baseline and follow-up 1, with no statistically significant change between follow-up 1 and follow-up 2 (Table 4).

**Table 4:** Changes of clinical and MR-derived data during follow-up.

	Group 1—all patients (n = 13)		Group 2—patients who participated in FU2 (n = 7)			
	baseline	FU1 vs. baseline	baseline	FU1 vs. baseline	FU2 vs. baseline x	FU2 vs. FU1 x
6MWT [m] *P* value	503 ± 126	-53 ± 46 **0.006**	508 ± 133	-59 ± 54 **0.043**	-40 ± 82^c^ 0.345	6 ± 25 0.893
4-step [s] *P* value	3.1 ± 2.4^a^	1.2 ± 2.2 **0.034**	3.5 ± 3.3^b^	1.4 ± 2.8^b^ 0.075	0.4 ± 0.7^c^ 0.465	0.2 ± 0.7^c^ 0.715
FVC_sit_ [l] *P* value	2.9 ± 0.7	0.2 ± 0.6 0.328	2.7 ± 0.7	0.3 ± 0.6 0.398	0.1 ± 1.1 1.0	-0.2 ± 0.7 0.31
FVC_sup_ [l] *P* value	2.5 ± 0.7	0.1 ± 0.8 0.972	2.5 ± 0.6	0.2 ± 0.7 0.735	0.1 ± 1 0.31	-0.1 ± 0.5 0.31
CK [U/l] *P* value	785 ± 273	-209 ± 224 **0.016**	848 ± 215	-359 ± 161 **0.018**	-424 ± 186 **0.018**	-65 ± 70 0.063
FF_psoas_ *P* value	0.36 ± 0.37	0.05 ± 0.09 **0.016**	0.23 ± 0.24	0.04 ± 0.06 0.091	0.1 ± 0.14 0.063	0.06 ± 0.14 0.499
FF_para_ *P* value	0.56 ± 0.39	0.03 ± 0.1 0.753	0.47 ± 0.41	0.06 ± 0.14 0.866	0 ± 0.08 0.735	-0.06 ± 0.1 0.176
Mercuri_psoas_ *P* value	2.1 ± 1.3	0.2 ± 0.4 0.083	1.6 ± 0.8	0.3 ± 0.5 0.157	0.6 ± 0.8 0.102	0.3 ± 0.5 0.157
Mercuri_para_ *P* value	2.7 ± 1.4	0.2 ± 0.4 0.083	2.3 ± 1.4	0.4 ± 0.5 0.083	0.4 ± 0.5 0.083	0 ± 0 1.0

Baseline values and changes versus the baseline (described as mean ± standard deviation) are shown for patients with one (group 1), and two (group 2) follow-up MR examinations, respectively.

^a^ n = 12

^b^ n = 6

^c^ n = 5

FU = follow-up

6MWT = six-minute walk test

4-step = Four-step stair climb test

FVC_sit_ = forced vital capacity in the sitting position

FVC_sup_ = forced vital capacity in the supine position

CK = creatine kinase

FF_psoas_ = MR-derived fat fraction of the psoas muscle

FF_para_ = MR-derived fat fraction of the paraspinal muscles

**Fig 3:**
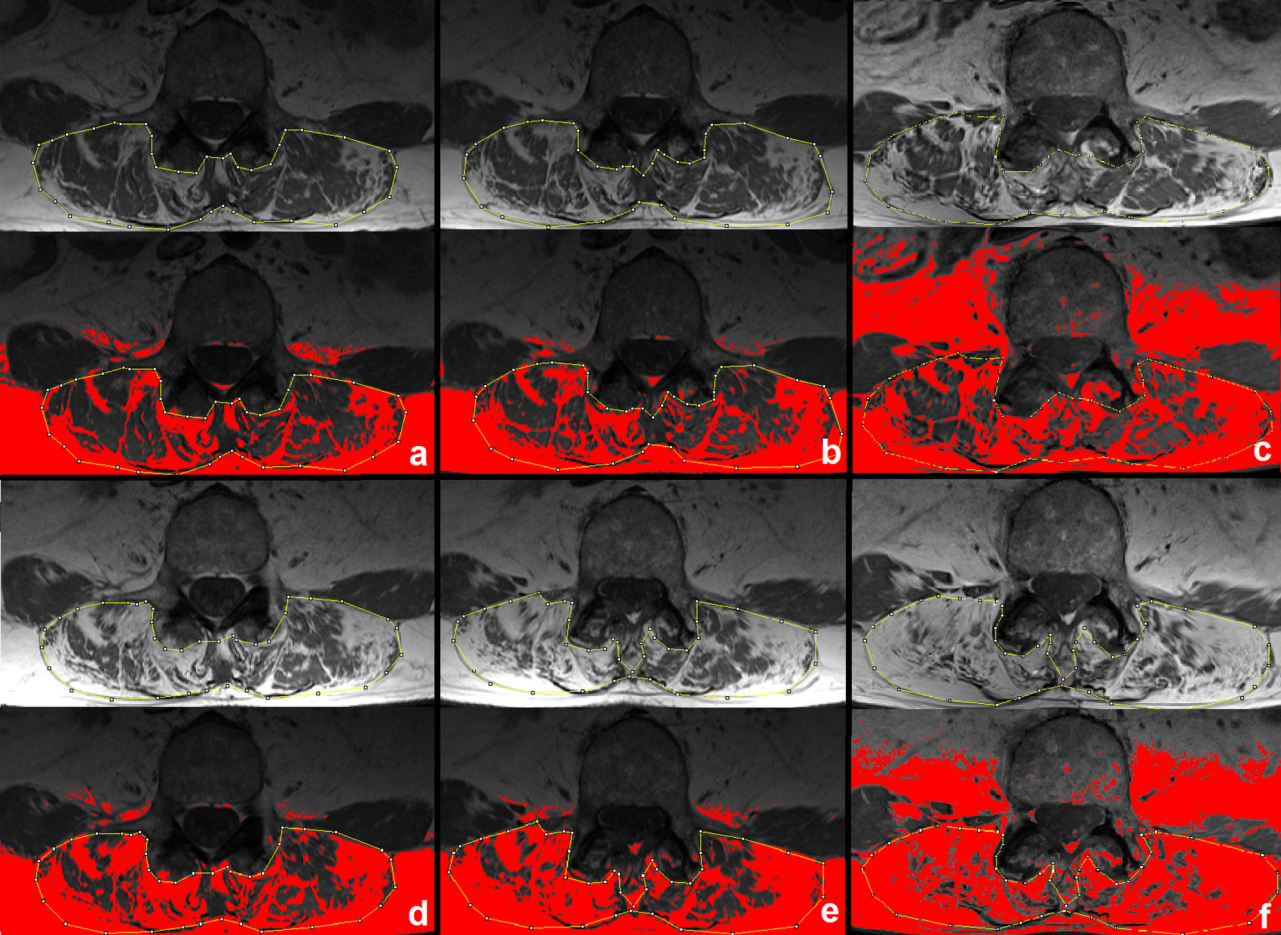
Fat fraction of the paraspinal muscles during follow-up. Images acquired at baseline (panel a), followed-up yearly (panel b-f) are shown. Fatty infiltrated muscle is colored red. The fat fraction increased from 0.38 (panel a) via 0.46 (b), 0.53 (c), 0.57 (d), 0.66 (e) to 0.74 (f).

Additionally, one patient who did not receive enzyme replacement therapy because of age (70 years at the time of initial diagnosis) was followed-up yearly to demonstrate the value of the proposed quantification method. In this patient, the psoas muscle was already completely degenerated at the initial presentation, but the FF of the paraspinal muscle had increased linearly from 0.38 at baseline to 0.74 at 5 years (Fig 3).

## Discussion

In our study, we evaluated a simple method to quantify muscular degeneration and replacement by fat based on commonly used (turbo) spin echo MR-sequences. This method, previously used for degenerative spine diseases like symptomatic disc herniation with lower back pain [25], was applied on a large collective of patients with LOPD, using conventional T1-weighted images. A high inter-observer agreement, in addition to previously reported high intra-observer reliability [19,26], as well as good correlations with clinical data were demonstrated. This simple type of measurement would easily translate to the clinic, with the added advantage that, compared to Mercuri scoring, the relatively wide intervals of FFs in a given Mercuri score are expressed with a percentage-based and therefore more precise scale (Fig 2).

A literature review revealed inconsistency about whether T1- or T2-weighted images are preferred when assessing fatty muscle degeneration by conventional MR sequences. While the majority of authors prefer T1-weighted sequences [13,17,27,28], others have used T2-weighted images [19,25]. T2-weighted imaging risks overestimating fatty muscle degeneration because fat, edema, and high glycogen content appear hyperintense; thus, T1-weighted images would be preferable to monitor Pompe disease. Differences in FF between the L3- and L5-levels were very low in absolute numbers. These results suggest that the mean value of several slices covering the whole muscle volume should be calculated to generate an overall score.

The main technical limitation when using TSE sequences for fat detection is the presence of field inhomogeneity causing high signal intensity variation, which aggravates with increasing field strength and has been reported to be up to 80% at 3 T [29]. Additionally, the method requires a clear separation of muscle and fat, which is far from being guaranteed in fat infiltrated muscles, with possibly a large range of voxel FF values.

Field inhomogeneities account for high differences in the signal intensity of subcutaneous fat, which was used for reference by Mhuiris et al [18]. This is the main limitation of their method, especially because the ROIs used in their study were very small. In contrast, the method used here smoothens field inhomogeneities by the definition of an intra-individual threshold in the area of interest, and subsequent binary assignment of a single voxel to fat or muscle.

In recent studies, the quantification of intramuscular fat has been achieved using Dixon water and fat separation techniques [15,28] or T2-quantification [30]. For example, excellent inter-observer and retest accuracy in patients with limb girdle muscular dystrophy 2I have been demonstrated [31]. However, the superiority of Dixon MR-sequences vs. conventional imaging is offset by their frequent retrospective unavailability in older imaging datasets. Still, those modern sequences should be used in prospective studies because of their higher accuracy in the differentiation of water and fat signals, voxel-by-voxel, which is mainly a result of their higher robustness against B0- and B1-inhomogeneities of the magnetic field [32].

Mercuri scores as well as FF values correlated well in the current study with MRC scores of muscle strength, agreeing with recent whole-body MRI studies in which MRI parameters were more sensitive in identifying early and clinically silent changes in the musculature [28,33]. Significant correlations between Mercuri scores and functional patient stage have also been published [34]. In our study, levels of plasma CK were low in patients with advanced disease (Mercuri 4), but comparatively high in patients with normal or mild muscle changes, as demonstrated by MRI (Table 3). This pattern confirms that MRI data are advantageous in detecting early changes in the musculature [28] and correlate with the clinical presentation of patients. Only moderate correlations were found between the Mercuri score or FFs and results of the clinical tests, indicating that more factors than assessable by lumbar muscle MRI (e.g., cardiopulmonary function, training level, and personal patient characteristics such as age and body weight) contribute to the clinical presentation of LOPD patients.

The main clinical use of our method was to assess long-term follow-up of LOPD patients under ERT. We found a significant decrease in plasma creatine kinase after ERT initiation. Clinically, a decrease in performance on the 6-minute walk test and 4-step stair climb test was demonstrated at follow-up 1 after a median of 39 months, which reached a plateau between follow-up 1 and follow-up 2 after a median of 63 months. Of interest, a comparable trend was seen for the MR-derived FF of the psoas, but not for the paraspinal muscles, indicating that especially the psoas (in addition to limb muscles) should be taken into account when assessing disease severity in prospective studies. A recent meta-analysis has shown that performance on the six-minute walk test usually increases after initiation of ERT and stays stable afterwards [7]. However, follow-up times are short in most included studies (ranging from 3 to 75 months), and only one study had a follow-up of more than 3 years [7,35]. Although it is an objective clinical test, the 6-minute walk test can involve high performance variability depending on external or internal conditions [21]. Therefore, other parameters such as those derived from MRI might increase objectivity to evaluate treatment response. Additionally, compared with other studies [5,36–38], patients in our study performed better on the 6-minute walk test at baseline (mean walking distance was 503 m in our study, versus 341 m [37], 332 m [5], 265 m [36], and 246 m [38], respectively), indicating a milder disease severity. This difference might explain the fact that despite ERT administration, performance deteriorated until follow-up 1. In addition, an increased performance in the 6-minute walk test can be supported by physical activity. For example, in the study by Strothotte et al [37], patients with the highest improvements regularly performed training programs. Participation in a physical training program was recommended to all patients included in our study. However, as these programs were not centrally organized, no data concerning the results of these programs, or frequencies of participation were assessed. Slowly increasing fatty muscle degeneration under ERT has also been demonstrated by Carlier et al [15] using Dixon-based quantification of the FF; however, long-term results are as yet unavailable. In our study the FF remained constant after an initial increase, which may support the long-term effectiveness of ERT. However, this conclusion should be taken with caution, because the study was retrospective and no placebo-controlled or non-treated group was analyzed. Still, the patient who did not receive ERT showed a much faster fatty muscle degeneration as depicted by MRI (FF of the paraspinal muscles increased by 19% after 3 years of follow-up) compared with the 13 patients under ERT (mean FF increase at follow-up 1 was 3%; only 2/13 patients showed an increase of more than 19%). A similar tendency was not significantly depicted by applying the Mercuri score, which was expected, as a change in this score requires a substantial increase in FF, which is very unusual even in severe cases. Therefore, the Mercuri score seems to be not as appropriate for monitoring subtle muscle degenerative changes, as also recently shown in a longitudinal study of patients with LGMD2I [31].

### Strengths and limitations

Study limitations include the retrospective design, with examinations performed on multiple MR scanners with different field strengths, and the fact that the number of patients examined on a 3 T scanner was low. Because field inhomogeneity increases at 3T and no correction filter for signal non-uniformity was used, a further validation of the method should be performed in future studies for 3 T scanners. Still, the main use of the method is the analysis of older imaging data, and these examinations have been most frequently performed on 1.5 T scanners. Additionally, the proposed quantification method could not be compared with Dixon-based or other contemporary quantitative techniques, because these data were not retrospectively available, and inter-study reliability was not tested. Also, the definition of a threshold for normal muscle tissue is influenced by the reader and therefore less objective than proton-density or Dixon imaging. Thus, automated approaches as recently proposed might increase objectivity in future studies [39]. In addition, the binary separation of every voxel being counted as either muscle or fat is a weakness of the method, whereas water/fat (Dixon) imaging methods allow for a precise measurement of the degree of fat infiltration, voxel by voxel [15]. However, semi-automated quantification of fatty muscular degeneration demonstrated, in particular, high inter-observer agreement (for a relatively large patient cohort), correlation with clinical data, ease of use, and a retrospective application to gauge disease progression (superior to Mercuri scoring). Another limitation is that correlates with clinical data were assessed using imaging data of lumbar muscles only, but these muscles–though less frequently assessed in the literature–are of high clinical relevance for LOPD patients, and their degeneration might lead to the initial clinical symptoms in these patients [40]. In addition, as the proposed methods could be applied to any specific muscle, the incorporation of upper leg muscle imaging might be of use in future studies. Finally, for a more detailed analysis, whole muscle volumes could be covered by using more slices, with the drawback of longer evaluation times.

## Conclusions

This retrospective monocentric study confirmed the value of muscle MRI to follow-up LOPD patients, and validated a simple and rapid method to retrospectively assess fatty muscle degeneration based on conventional T1-weighted TSE sequences. The following conclusions could be drawn:

- A rapid and simple quantification of fatty muscle degeneration can be achieved using conventional T1-TSE sequences with a high inter-observer agreement.
- This method can be used for a more accurate retrospective longitudinal evaluation of older imaging data compared with Mercuri scoring, when other sequences are unavailable. For prospective studies, modern water-/fat- (Dixon-) imaging should be used due to its robustness against B0-/B1- inhomogeneities of the magnetic field and more objective voxel-by-voxel calculation of the FF.
- MRI-derived muscle-specific data correlate with clinical parameters (6-minute walk test, forced vital capacity, MRC scores of muscle strength), and may be used to monitor ERT.
- After an initial increase, the FF of the psoas muscle stayed constant during long-term follow-up under ERT.

## Supporting information

S1 Table Characteristics of the 13 patients who underwent follow-up under enzyme replacement therapy. (DOCX)

S1 Dataset Anonymized raw dataset including baseline data of all 41 patients. (XLSX)

S2 Dataset Anonymized raw dataset including baseline and follow-up data of 13 patients under enzyme replacement therapy. (XLSX)
