# Supplementary material for: Quantification of intramuscular fat in patients with late-onset Pompe disease by conventional magnetic resonance imaging for the long-term follow-up of enzyme replacement therapy
Source: PLoS One. 2018 Jan 9;13(1):e0190784. doi: 10.1371/journal.pone.0190784 (PMC5760036; doi:10.1371/journal.pone.0190784)
Supplement: S1 Table — (DOCX) [file pone.0190784.s001.docx]

| **patient** | **sex** | **year of birth** | **onset** | **GAA mutation** | **BMI [kg/m²]**  **at T0/FU1/FU2** | **age [years]**  **at T0/FU1/FU2** | **use of walking device**  **at T0/FU1/FU2** | **use of respiratory support**  **at T0/FU1/FU2** | **WGMS**  **at T0/FU1/FU2** |
| --- | --- | --- | --- | --- | --- | --- | --- | --- | --- |
| **1** | female | 1982 | juvenile | c.2014C>T  c.1703A>T | 19.6 / 20.3 / 21 | 24 / 28 / 29 | no | no / no / BiPAP(s) | 3 / 3 / 4 |
| **2** | female | 1995 | juvenile | c.-32-13T>G  c.307T>G | 17.7 / 20.3 / 20.3 | 12 / 15 / 16 | no | no | 2 / 2 / 2 |
| **3** | female | 1947 | adult | c.-32-13T>G  c.2322_2323ins | 19.4 / 19.7 / n-a | 59 / 62 / n-a | no | no | 3 / 3 / n-a |
| **4** | male | 1993 | juvenile | c.-32-13T>G  c.-32-13T>G | 19.7 / 20.7 / 22.2 | 13 / 16 / 18 | no | no | 0 / 0 / 0 |
| **5** | female | 1959 | adult | c.-32-13T>G  c.525delT | 21.1 / 21.1 / 22.5 | 46 / 50 / 53 | no | no | 3 / 3 / 3 |
| **6** | male | 1962 | adult | c.-32-13T>G  1143delC | 26.3 / 26.6 / n-a | 47 / 50 / n-a | WC(p) / WC(p) / n-a | BiPAP(s) / BiPAP (s) / n-a | 3 / 4 / n-a |
| **7** | male | 1971 | adult | c.-32-13T>G  c.1291_1299del | 25.1 / 22.9 / 22.9 | 35 / 39 / 40 | no | no | 3 / 3 / 3 |
| **8** | female | 1962 | adult | c.-32-13T>G  c.877G>A | 26.6 / 27.2 / n-a | 47 / 51 / n-a | no / WC(p) / n-a | no | 4 / 4 / n-a |
| **9** | male | 2001 | juvenile | c.-32-13T>G  c.1561G>A | 14.7 / 15.2 / 15.8 | 6 / 9 / 10 | no | no | 0 / 0 / 0 |
| **10** | male | 1993 | juvenile | c.-32-13T>G  c.525delT | 15.2 / 16.5 / n-a | 15 / 18 / n-a | no | no | 0 / 0 /n-a |
| **11** | female | 1964 | adult | c.-32-13T>G  c.1291_1299del | 21 / 19.4 / 20.2 | 42 / 46 / 49 | no | no | 2 / 2-3 / 3 |
| **12** | female | 1999 | juvenile | c.-32-13T>G  c.1396G>T | 17.2 / 18.7 / n-a | 11 / 13 / n-a | no | no | 0 / 0 / n-a |
| **13** | male | 1977 | juvenile | c.-32-13T>G  c.2481+102_2646+31del | 24.9 / 25 / n-a | 29 / 32 / n-a | no | no | 3 / 3 / n-a |

**S1 Table. Characteristics of the 13 patients who underwent follow-up under enzyme replacement therapy.**

GAA = glucosidase alpha, acid

BMI = body mass index

T0 = baseline, initial MRI and first ERT infusion

FU1 = follow-up 1

FU2 = follow-up 2

WGMS = Walton & Gardner-Medwin scale

n-a = not applicable

WC(p) = wheelchair (partly)

BiPAP(s) = Biphasic Positive Airway Pressure (in the supine position)
